# New Cytokines in the Pathogenesis of Atopic Dermatitis—New Therapeutic Targets

**DOI:** 10.3390/ijms19103086

**Published:** 2018-10-09

**Authors:** Jolanta Klonowska, Jolanta Gleń, Roman J. Nowicki, Magdalena Trzeciak

**Affiliations:** 1 Military Specialist Clinic, Allergy Clinic, ul. Dąbrowskiego 1, 87-100 Toruń, Poland; 2 Department of Dermatology, Venereology and Allergology Medical University of Gdansk, ul. Kliniczna 1a, 80-401 Gdańsk, Poland; jglen@gumed.edu.pl (J.G.); rnowicki@gumed.edu.pl (R.J.N.); mtrzeciak@gumed.edu.pl (M.T.)

**Keywords:** atopic dermatitis, cytokines, IL-17, IL-19, IL-33, TSLP, biological agents

## Abstract

Atopic dermatitis (AD) is a recurrent, chronic, and inflammatory skin disease, which processes with severe itchiness. It often coexists with different atopic diseases. The number of people suffering from AD is relatively high. Epidemiological research demonstrates that 15–30% of children and 2–10% adults suffer from AD. The disease has significant negative social and economic impacts, substantially decreasing the quality of life of the patients and their families. Thanks to enormous progress in science and technology, it becomes possible to recognise complex genetic, immunological, and environmental factors and epidermal barrier defects that play a role in the pathogenesis of AD. We hope that the new insight on cytokines in AD will lead to new, individualised therapy and will open different therapeutic possibilities. In this article, we will focus on the cytokines, interleukin (IL)-17, IL-19, IL-33, and TSLP (thymic stromal lymphopoietin), which play a significant role in AD pathogenesis and may become the targets for future biologic therapies in AD. It is believed that the new era of biological drugs in AD will give a chance for patients to receive more successful treatment.

## 1. Introduction

Atopic dermatitis (AD) is a chronic, inflammatory skin disease which is characterized by severe itchiness. It affects 15–30% of children and 2–10% of adults [1] seriously decreasing the quality of their life [2]. In recent years, special attention has been paid to immunological factors of Atopic dermatitis (AD) pathogenesis, in addition to epidermal barrier defects. They include numerous disorders of Th2 lymphocytes and the cytokines released by them, IL-4, IL-5, IL-13, and lead to elevated production of IgE, increased inflammation in the skin, and aggravate the skin barrier defect in AD [3]. In addition to the Th2-dependent response, the influence on inflammation in the skin of patients suffering from atopic dermatitis exerts well-known Th2 lymphocytes, also Th17 and Th22 lymphocytes releasing, among others, such cytokines as: IL-17, IL-19, and IL-22 [4,5]. The response of T lymphocytes and the domination of cytokines secreted by them differs significantly in the stage of AD exacerbation and in the remission period [3,4]. Th2 lymphocytes (IL-4, IL-13, IL-31), Th1 and Th22, are active in patients with external and intrinsic AD. However, Th17 and Th9 lymphocytes or cytokines IL17, IL12/IL23, and IL9 predominate in patients with intrinsic AD. Ethnic differences in the profiles of lymphocytes and cytokines are also observed. Thus, Asians with AD, even in the presence of elevated serum IgE concentration, while maintaining a strong component of Th2 cells, are characterized by a greater activation of Th17 and Th22 lymphocytes (IL17A, IL19, and IL22) in altered and unchanged skin compared to Europeans with AD [5]. In addition, keratinocytes under the influence of various factors, such as exposure to allergens, microbial action, scratching resulting from pruritus—the main symptom of AD, react by releasing cytokines important for inflammation, including TSLP (thymic stromal lymphopoetin), IL-33, and IL-25. IL-33 activates Th2 lymphocytes and congenital lymphoid cells (ILC2). In turn, ILC2, together with IL-33, IL-25, and TSLP, seem to explain and differentiate between the mechanism of atopic march from development and the epidermal barrier defect [6,7].

The multifactorial background of AD explains therapeutic failures, justifies the tendency to therapy optimisation in accordance with pathogenesis, the need for individualization of the treatment, and the search for new solutions. It is suggested that based on various characteristics, e.g., patient age, the onset of the disease, disease severity, triggers, response to therapy, biomarkers, genetic variants, and immunological polarization, different subtypes of AD may be distinguished (phenotypes, endotypes, genotypes, immunotypes) [8]. Subtypes’ definition may be used to select new directions of clinical trials and to develop therapies for patients who will benefit from the treatment based on targeted immunological mechanisms.

**Figure 1:**
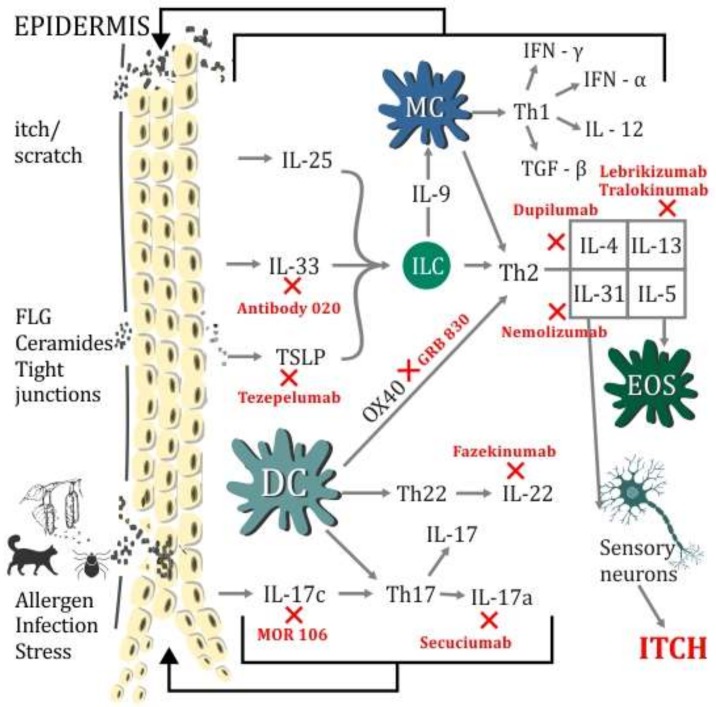
Schematic summary of immunological disorders in Atopic dermatitis (AD) pathogenesis coexisting with skin barrier defect. The diagram shows inflammatory cells, Th2, Th17, and Th22-dependent inflammation in AD with cytokines, which diminish the epidermal barrier. The impact of infections, allergens, stress, and itchiness, leading to the activation of inflammatory pathways. The figure depicts the possible targets of biological agents in AD treatment. DC (dendritic cells), EOS (eosinophil), FLG (filaggrin), IL (interleukin), IFN-α (interferon-alfa), IFN-γ (interferon gamma), ILC (lymphoid cells), MC (mast cells), TGF-β (transforming growth factor beta), TSLP (thymic stromal lymphopoietin). X—indicates potential areas of new biological drugs action.

In this article, we will take a closer look at new cytokines: IL-17, IL-19, IL-33, and thymic stromal lymphopoietin, whose role in the development of AD and probably other atopic diseases is gaining importance. These cytokines give hope in the field of pathogenesis, and the search for potential genetic/molecular/biological markers among them. This work will also indicate the potential area of these cytokines in the treatment of AD in the future (Figure 1).

## 2. TSLP—Thymic Stromal Lymphopoietin

The thymic stromal lymphopoietin was discovered 20 years ago as a secretory factor of thymic stromal cells in mice. The gene encoding TSLP in humans is found on the chromosome, 5q22.1, in addition to the genes grouped on the 5q31 chromosome encoding the known Th2-dependent cytokines: IL-4, IL-5, and IL-13. TSLP is a cytokine that uses the combination of JAK1 and JAK2 to essentially activate STAT5 proteins [9]. TSLP comes from epithelia/epithelium and fulfills its biological function through the TSLP receptor (TSLPR) [10]. TSLPR is present on dendritic cells (DC), T and B lymphocytes, NK cells, ILC2, eosinophils (EOS), basophils, and monocytes [11,12,13]. TSLP strongly activates immature dendritic cells, increasing the expression of CD80, CD86, and OX40L molecules and the production of chemokines, and inhibits IL-12 production. In this way, TSLP stimulates the Th2 response [14]. TSLP is responsible for the maturation of antigen presenting cells (APC). It promotes the activity and chemotaxis of eosinophilia [15]. It enhances the expression of IL-4, IL-5, and IL-13 in IL-33 stimulated human ILC2 cells [16] and activates ILC2 in the lungs and skin of mice [12,13]. TSLP and IL-31 stimulate sensory skin neurons involved in the pathomechanism of pruritus. TRPA1 (transient receptor potential) is required for TSLP-induced activation of sensory neurons that lead to pruritus. TSLP released from keratinocytes acts directly on sensory neurons [17].

Studies on mouse models suggest the role of TSLP from keratinocytes in the development of allergic airway inflammation [18,19,20,21]. Skin allergen exposure in the presence of TSLP may trigger progression from atopic dermatitis to asthma. In the mouse model, TSLP and OVA (Ovalbumin) applied intradermally were the reason why mice sensitized to this allergen, and developed allergic inflammation in the airways after inhalation challenge with the same allergen. The results indicate that TSLP only participates in the sensitization phase, whereas the response to inhalation challenge is already independent of TSLP [22]. In another experimental model, TSLP stimulation increased the probability of developing food allergy by inducing sensitization to allergens through the skin [23]. These data seem to explain the molecular mechanism of the allergic march development [7].

## 3. TSLP in AD (Atopic Dermatitis)

It has been demonstrated that the concentration of TSLP in blood serum, in both children and adults with AD, is significantly increased compared to healthy people [24,25]. In addition, higher cytokine expression was demonstrated in keratinocytes of patients with AD, which correlates with the severity of the course of the disease and impaired function of the epidermal barrier measured by the degree of hydration of the stratum corneum and transdermal water loss. The use of moisturizing substances reduces TSLP levels and reduces the symptoms of AD and the severity of the disease course [26]. The increase in TSLP production in keratinocytes is affected by both physical epidermis (scratching, tape-stripping) and Staphylococcus aureus, which colonizes the skin in 90% of AD patients [26,27,28,29,30].

The polymorphism of the gene coding for TSLP has been associated with the risk of the development and course of AD. The carriers of the genotype, CC, of the TSLP gene, rs2289278, showed an increased risk of developing AD. The correlation was stronger in atopic children than in children without atopy. In addition, the C rs2289278 allele significantly increased the risk of developing asthma in children with AD. [31]. Gao et al. assessed the variability of TSLP as a risk factor for the development of atopic dermatitis and diagnosis of herpetic eczema (ADEH) in people with AD. They noticed that the polymorphism of the TSLP gene, rs1898671, was associated with a reduced risk of herpetic eczema [32]. Margolis and colleagues found in the studies that this genetic variant of TSLP rs1898671 was associated with a reduced likelihood of a persistent form of AD and it did not cause any additional risk of asthma [33]. In addition, these researchers demonstrated that people with FLG gene loss mutation, who are more likely to have severe forms of AD, if they had the TSLP genetic variant rs1898671, they were nearly five times less likely to have persistent AD in comparison to those without this TSLP genetic variant [33]. Subsequent studies indicate that the coexistence of the TC and CC genotypes in the TSLP encoding gene, rs1837253, may be associated with eczema. In women with these genotypes, compared to carriers of the TT genotype (rs1837253), the risk of eczema significantly increased with age and patient smoking. In the analysis, however, this relationship was not statistically significant. No relationship was found between SNPs (Single Nucleotide Polymorphisms) rs3806933 or rs2289276 and eczema [34]. However, it was shown that the polymorphism of the TSLP encoding gene (rs1837253) can be directly involved in the regulation of TSLP secreted from the cell [35]. Thus, the TSLP genetic variants may result in a decrease in the expression and activity of the TSLP protein that gives a protective effect for the development of AD and allergy [33]. The existence of polymorphisms in the TSLP coding gene was also associated with the risk of developing AD [31] or asthma [36].

Taken together, TSLP is a characteristic promoter of atopic inflammation, leads to a chronic Th2 inflammatory response, and plays a key role in chronic atopic diseases, which means that it is a promising pharmacological goal.

## 4. Anti-TSLP Therapy, Anti OX40

Tezepelumab (formerly referred to as MEDI9929, previously AMG-157) is an antibody directed against circulating TSLP. The examination of a Phase IIa, randomized, double-blind, placebo-controlled (RDBPC) study with thezepelumab aimed to evaluate the efficacy and safety of MEDI9929 in 155 adults with moderate to severe AD. Participants received six doses of MEDI9929 (280 mg) subcutaneously or placebo every two weeks for 12 weeks (with the last dose in week 10). All participants were monitored until the 22nd week. Unfortunately, the results have not yet been published (NCT02525094) [37].

In a subsequent study, patients with moderate to severe AD received an intravenous formulation against TSLP, here called MK-8226. The safety, tolerability, efficacy, pharmacokinetics (PK), pharmacodynamics (PD), and immunogenicity of MK-8226 were assessed. The study was completed earlier due to business reasons. The final results from the analysis were summarized, not published [38]. The GBR 830 monoclonal antibody is an OX40 antagonist. A phase 2a study lasting 12 weeks was used in the RDBPC. EASI 50 reached 23 of 17 treated patients, and the remaining data is under analysis [39].

## 5. Interleukin-33

IL-33 belongs to the IL-1 superfamily, the alarmin family. It is secreted by macrophages, dendritic cells, fibroblasts, adipocytes, smooth muscle cells, endothelial cells, bronchial epithelium, osteoblasts, and the intestines [40,41,42,43,44] after cell damage signal. IL-33 attaches to a specific Toll receptor (TLR)/IL1R superfamily, i.e., the ST2 receptor, which forms a heterodimer with the IL-1 receptor-associated protein (IL-1RAcP), initiating the immune cascade [45]. A very important step in ligand binding and IL-33 activity is the heterodimerization of the ST2 receptor. This receptor may be present within the cell in two isoforms, as a membrane or transmembrane form (ST2 or ST2L) and a secreted or soluble form (sST2), which is also called an endogenous form. The transmembrane form of ST2 is mainly expressed on congenital lymphoid 2 (ILC2) cells, mast cells, basophils, dendritic cells, NK cells, and Th2 lymphocytes [43,44,45]. IL-33 binds to the ST2 or IL-1RAcP receptors, paves the signal transduction pathway, and engages MyD88 (myeloid differentiation primary response gene 88) and a number of kinases: Interleukin receptor associated kinase (IRAK) 1/4, IRAK 1/2, p38 MAPK, and JNK [41,45,46,47]. It stimulates the NF-κB transcription factor and the production of dependent Th2 cytokines. The main MyD88 molecule (adapter protein) is essential for IL-33 for the production of Th2 cytokines and mast cell proliferation and degranulation [48,49]. IL-33 activates mast cells (MC) and basophils, and induces overproduction of proinflammatory cytokines synthesized by these cells. In addition, it also causes migration, maturation, adhesion, and survival of these immune cells [50,51,52]. The impact of genetic variants of IL-33 and its receptors on the risk of asthma and/or allergy in humans was investigated.

The results of studies carried out in the Brazilian population demonstrate a strong relationship between the genetic variants of the gene encoding IL-33 and IL1RL1 with allergy and asthma markers. The G allele of the polymorphism of the IL33 gene, rs12551256, showed a negative correlation with asthma, whereas the A IL1RL1 gene allele, rs1041973, correlated with the production of IL-5 in the serum, sIgE levels, and positive scores of skin prick tests [53].

The results of a study in the Chinese population indicated a statistically significantly higher frequency of the G allele of the polymorphism of the IL33 gene, rs928413, and the C allele of the polymorphism of the IL1R1 gene, rs6871536, in patients with asthma, and confirms the relationship between the studied polymorphisms and the development of asthma. [54].

Subsequent studies on polymorphism of the IL-33 gene, rs928413, rs1342326, indicated their association with hay fever in the population of six-year-old children [55].

Published research results encourage the study of a possible association of IL-33 gene polymorphisms with the risk and severity of atopic dermatitis in the Polish population.

## 6. IL-33 in AD

In experimental studies on the mouse models of hK12mIL-33tg, it was revealed that for skin-like AD symptoms similar to the accumulation of eosinophils and mast cells, excessive expression of IL-33 should be blamed [56]. In addition, increased expression of IL-33 and ST2 in the skin of mice, which was characterized by filaggrin (FLG) deficiency, was discovered. This could indicate the relationship between IL-33 and the epidermal barrier defect in AD, which requires further study [57]. On the basis of the overexpression of IL-33 in the epidermis and infiltration of ST2-positive cells in patients with AD, it was found that the IL-33/ST2 pathway plays an important role in the pathogenesis of AD [58]. IL-33 stimulates ILC2 cells. Their expression is clearly elevated in AD and increases after allergen exposure [59,60]. Other studies have shown that patients with AD have significantly elevated serum levels of TSLP, IL-31, and IL-33. The level of IL-31 and IL-33 in the serum of patients with AD was higher in children than in adults, whereas in the case of sST2, this was inverse. A positive correlation was found between TSLP, IL-31, and IL-33 as well as an inverse relationship between IL-33 and sST2 [24]. In turn, the use of monoclonal antibody 020-against IL-33 (Figure 1), in the phase 2a of the study, where itching was assessed, SCORAD, DLQI, IGA, and EASI allowed a rapid response to a single dose of the drug to be obtained, and all patients achieved EASI 50 within 57 days [61].

## 7. Interleukin IL-17

In 2000, T-lymphocytes producing IL-17 were first recognized as a Th-subtype of a Th-cell subtype distinct from Th1 and Th2, expanding the Th1/Th2 dichotomy in allergy to other effector cell subtypes [62]. The distinctive feature of Th17 lymphocytes is the ability to produce IL-17 and negative regulation by IFN gamma (IFN-γ) as well as IL-4 [63,64]. The formation of Th17 lymphocytes is mainly stimulated by TGF-beta (transforming growth factor-beta; TGF-β), IL-6 as well as IL-1beta, IL-7, IL-21, and IL-23 and is inhibited by IL-27 and the cytokines of Th1 cells (IFN-γ) and Th2 (IL-4, IL-5) [65,66].

IL-17 has been described as a cytokine secreted by Th17 lymphocytes and recently discovered ILC cells. ILCs are effector cells of innate immunity. ILC2 was detected in the skin, peripheral blood, gastrointestinal tract, and airways. ILC can be divided into three groups based on the dominant type of cytokines. ILC1 produce Th1 cytokines (including IFN-γ), ILC2 produce Th2 cytokines (IL-5, IL-13); and ILC3 of Th17 cytokine (IL-17, IL-22). The synthesis of IL-17 is strongly stimulated in chronic autoimmune processes, atopic dermatitis, and asthma. The IL-17 family of cytokines is a group of homologous proteins: IL-17A and IL17B to IL17F [67,68,69]. Genes for IL-17A and IL-17F are on the same chromosome—6p12. The genes of other IL-17 family cytokines are located on different chromosomes: IL-17B-5q32-34, IL-17C-16q24, IL-17D-13q12.11, and IL-17E (IL-25)-14q11.2. IL-17A-IL-17F cytokines possess proper receptors, which, after being activated, induce inflammatory processes. [69,70].

IL-17C is a unique cytokine, a functionally separate member of the IL-17 family involved in inflammation enhancement in both psoriasis and AD. It comes from epithelial cells/keratinocytes and is also induced thorough TLR2 and TLR5 by bacteria [71]. IL-17C acts thorough the receptors, IL-17RA and IL-17RE, localized on different cells, like lymphocytes T and keratinocytes [72]. It stimulates Th17 T cells to begin IL-17A/F and IL-22 production. The mutual cooperation between IL-17A and IL-17C was observed [72].

### 7.1. IL-17 in AD

Studies prove the importance of IL-17 in the pathogenesis of AD. In the mouse AD model, the Th2 response was shown to be regulated by IL-17. The lack of IL-17A reduced dermatitis and IL-4 production as well as IgE production, and its presence triggered the production of IL-4 by Th2 cells [73]. After the stimulation of B lymphocytes by IL-17A, cells show prolonged survival and increased proliferation [74]. IL-17 promotes the differentiation of B cells to IgE producing plasma cells [56,75]. IL-17 initiates the production of some cytokines, IL-8, TNF-α, and TSLP; chemokines, CCL17 and CXCL10; and antimicrobial peptides [55,76,77]. It modulates the Th2 cellular immune response [57] and leads to the development of chronic AD phase [77]. It also deepens the defect of the epidermal barrier by inhibiting the expression of filaggrin in the skin of patients with AD [57,78]. It disturbs the regulation of genes encoding adhesion molecules of keratinocytes, including integrins, E-cadherin, and proteins involved in creating a physical epidermal barrier—claudins.

In the Koga et al. study, the number of Th17 cells in the peripheral blood and skin lesions of patients with AD was assessed, the effect of IL-17 on the production of cytokines/chemokines, and vascular endothelial growth factor (VEGF) by keratinocytes was investigated. The results suggest that the number of Th17 cells is elevated in blood and they may act as an enhancer of cutaneous lesions in AD. Immunohistochemical studies revealed infiltrates of IL-17 producing cells in diseased skin. Th17 infiltrates much more in acute lesions than in chronic ones depending on the severity of the lesions [79].

The IL-17A polymorphism was investigated in the aspect of the presence of atopic dermatitis in the Polish population. Narbutt et al. showed that despite the lack of an influence of the polymorphism -152G/A IL17A on the prevalence of AD in Poland, there is a significant correlation between the A/A genotype in -152G/A IL17A and the coexistence of AD and asthma. In addition, people with this genotype showed a higher risk of moderate and severe AD development [80].

Researchers from India showed a positive correlation of the AA variant IL-17F, rs1887570, with the number of sensitizing allergens in patients with asthma [81]. Expression of IL-17C was increased in lesional AD skin [82].

In the conducted studies, anti-IL-17C-MOR106 antibody was able to inhibit both Th2 and Th17/Th22 cells. The anti-IL-17C antibody, MOR106, which strongly and selectively binds to human and mouse IL-17C, inhibits the binding of IL-17C to the IL-17RE receptor. The antibody inhibits dermatitis induced by IL-23 in a model of psoriatic dermatitis. In diseased skin AD patients, the level of IL-17C expression in keratinocytes was increased. MOR106 has been tested in two different in vivo models. In the AD model of calcipotriol-induced dermatitis, the production of TSLP and IL-33 proteins was inhibited by MOR106. Consequently, in the mouse model with a flaky tail, the spontaneous development of AD-like skin inflammation was reduced by MOR106. In addition, serum IgE levels, number of mast cells in the skin, and IL-4 and CCL17 in serum were reduced. The results indicate that IL-17C plays an important role in AD, apart from psoriasis [82]. Interleukin 17C was also detected in the lungs and tissues of the skin after Mycoplasma pneumoniae and S. aureus infections [83].

Therefore, the therapy directed against IL-17A in patients with AD may inhibit Th2-dependent cytokine inflammation, reduce IgE levels, and lead to reduction of epidermal barrier dysfunction [57], and suppress not only Th2, but also Th17/Th22, response against IL-17C.

### 7.2. Anti-IL-17 Therapy

#### 7.2.1. Secukinumab

Secukinumab is a monoclonal antibody directed against IL-17A, registered for the treatment of psoriasis, psoriatic arthritis, and ankylosing spondylitis. In light of reports suggesting that the intrinsic type of AD is characterized by the domination of Th17 and Th9 lymphocytes with domination of the corresponding cytokine profile (IL-17, IL-12/IL23p40, IL-9) [84], suggestion of the Th17/Th22 endotype dependent on skin apparently unchanged in AZS [8], and ethnic differences indicating that the lymphocytic-cytokine profile in Asians with AD patients outside Th2 includes Th17, it seems that the subgroup of AD patients characterized by low levels of IgE and increased activation of Th17 can successfully respond to anti-IL17 therapy [84]. A single phase II trial was conducted—the evaluation of secukinumab in patients with AD. This is a randomized double-blind, pilot study of 44 patients divided into two groups: 22 patients with an internal and 22 with an extraneous form of AD [85].

#### 7.2.2. MOR106

MOR106 p/monoclonal antibodies against IL-17C were evaluated in the RDBPC clinical trial, phase 1 in 25 patients with AD. EASI-50 in the fourth week of therapy was achieved by 83% of patients. The drug worked quickly and the effects lasted for over two months [85].

## 8. Interleukin-19

Interleukin-19 (IL-19) is another pro-inflammatory cytokine that probably stimulates the production of Type 2 T-helper cells (Th2) [86]. Its gene is on chromosome 1q32. IL-19 belongs to the IL-10 family of cytokines (similar to IL-20,22, 24, and 26) [87]. Under the influence of IL-17A, IL-19 is strongly expressed in AD lesional skin. This suggests that IL-19 may be important for linking Th17 with Th2 in AD [86]. Thus, IL-19 seems to be interesting in the search for new drugs that would provide better disease control in patients with AD. Until now, single nucleotide polymorphisms in IL-19 coding genes have been investigated in relation to RSV (respiratory syncytial virus) infections and lower respiratory tract infections in term-born infants with subsequent episodes of wheezing [88].

## 9. Conclusions

The complex pathogenesis of AD increases the need to individualize treatment, which would allow increases in the effectiveness of therapy. Progress in science leads to more and more effective, selected AD therapies. After many years of research, the first biological drug, monoclonal antibody against IL-4R (Dupilumab), was registered, declaring a breakthrough in the treatment of AD. The results of clinical trials with the use of monoclonal antibodies against IL-13, IL-31 are promising. Will the drugs blocking the action of TSLP, IL-33, IL17A, and IL-19 ultimately play a role in the treatment of patients suffering from AD? Looking through the prism of pathogenesis, it seems that yes, but time, practice, and clinical research will verify it.
